# Clinical efficacy of multigene panels in the management of congenital hypothyroidism with gland in situ

**DOI:** 10.1097/MD.0000000000038976

**Published:** 2024-07-19

**Authors:** Jisun Park, Eun Young Joo, Myung Ji Yoo, Su-Jin Kim, Woori Jang, Ji-Eun Lee

**Affiliations:** a Department of Pediatrics, Inha University Hospital, Inha University College of Medicine, Incheon, Republic of Korea; b Gyeonggi-Incheon Regional Rare Disease Specialized Institution, Incheon, Republic of Korea; c Department of Clinical Laboratory Medicine, Inha University Hospital, Inha University College of Medicine, Incheon, Republic of Korea.

**Keywords:** congenital hypothyroidism, genes, levothyroxine, next-generation sequencing, precision medicine

## Abstract

Congenital hypothyroidism (CHT) is a diverse condition with various genetic etiologies. This study aimed to investigate the utility of next-generation sequencing (NGS) analysis in guiding treatment decisions and predicting prognosis for CHT patients with gland in situ (GIS). A retrospective analysis was conducted on 33 CHT patients with GIS who underwent NGS analysis at a single institution between 2018 and 2023. Patients were classified as having permanent (PCH), transient congenital hypothyroidism, or ambiguous congenital hypothyroidism (ACH) CHT based on their response to levothyroxine discontinuation at 3 years of age. Among the 33 patients, genetic variants were identified in 26, with the most prevalent variants found in *DUOX2* (26.92%), *TSHR* (30.77%), *TG* (19.35%), and *DUOXA2* (19.23%). Patients with high initial thyroid-stimulating hormone levels (>50 mIU/L) and low free thyroxine levels (<0.89 ng/dL) at diagnosis tended to have compound heterozygous or homozygous variants in *DUOX2*, *DUOXA2*, and *TG*, and were more likely to develop PCH. In contrast, patients with heterozygous variants in these genes often exhibited ACH. *TSHR* variants were associated with diverse clinical manifestations, ranging from PCH to ACH, and were more common in patients with initial thyroid-stimulating hormone levels <50 mIU/L. The study highlights the potential utility of NGS analysis in predicting the clinical course and guiding treatment decisions for CHT patients with GIS. Genetic analysis may aid in determining the appropriate duration of levothyroxine therapy and monitoring strategies, particularly in cases where traditional clinical indicators are inconclusive.

## 1. Introduction

Over the last 30 years, neonatal screening programs for the early detection of congenital hypothyroidism (CHT) in infants have been conducted, leading to successful treatment and prognosis of CHT.^[1]^ As diagnostic technology has advanced, the number of patients with CHT has increased, and efforts to identify the genetic etiology of CHT have continued. Primary CHT has diverse causes, including thyroid dysgenesis (TD), dyshormonogenesis (DH), thyroid-stimulating hormone (TSH) resistance, thyroid hormone transport or action defects, central hypothyroidism, and transient neonatal hypothyroidism, with TD being the predominant cause (85%) and DH contributing to 10% to 15% of cases.^[2,3]^

Advances in genetic analysis methods since the 2010s have enabled the assembly of next-generation sequencing (NGS) panels associated with CHT. These panels enabled a rapid and comprehensive evaluation of relevant genes from multiple perspectives, including gene dosage, copy number variations, and structural rearrangements. The introduction of NGS panels has transformed the diagnostic approach for CHT, providing deeper insights into the underlying genetic causes and suggesting the way for personalized treatment strategies.^[4,5]^ Researches have shown that DH in CHT is closely associated with genetic variants, particularly in genes such as *SLC5A5*, *TPO*, *DUOX2*, *DUOXA2*, *SLC6A4*, *TG*, *IYD/DEHAL1*, and *TSHR*.^[3,6,7]^ Among them, *TSHR* is known to be involved not only in TD and DH but also in TSH resistance in the thyroid gland in situ (GIS).^[8]^

The prevailing understanding is that most cases of DH caused by genetic variants are hereditary, in an autosomal recessive manner, and manifest as biallelic diseases.^[9,10]^ However, according to recent research results, hypotheses have been raised that genetic variants associated with DH, which have been known to follow an autosomal recessive inheritance pattern, could follow non-Mendelian mechanisms, such as autosomal monoallelic expression.^[7,11]^ Furthermore, hypotheses have been proposed regarding the mechanism of synergistic heterozygosity between various gene variants, each of which may lead to a cross-loss of enzyme activity.^[7]^ Indeed, this kind of hypothesis regarding the penetrance of the genotype variants or diverse phenotypes is really an interesting way to make a start with this study. Characteristic phenotypes based on the genetic variants have been reported. However, research examining treatment approaches for CHT based on genetic test results, particularly regarding medication discontinuation or maintenance and interpretation of blood test findings depends on the variants, has been limited. The authors anticipated that genetic analysis could emerge as a pivotal factor in guiding therapeutic decisions for congenital hypothyroidism.

The authors, therefore, summarized and reviewed the results of NGS analysis and clinical manifestations of CHT patients for 5 years at a single institution. Also, it was tried to reveal the manner in which genetic analysis could be supposed to affect the course of treatment and prognosis.

## 2. Materials and methods

### 2.1. Participants

The authors retrospectively examined the medical records and results of NGS analysis of patients diagnosed as CHT with GIS at Inha University Hospital between January 2018 and March 2023. The study included patients who had been diagnosed and treated for CHT at this institution or elsewhere. Eligible participants were those with a documented history of treatment, either ongoing or past, and had undergone NGS analysis. The initial diagnosis of CHT was determined based on the criteria of the 1997 National Newborn Screening Program in Korea, which includes an initial screening for elevated TSH levels in newborns, with a diagnostic cutoff value of 10 mIU/L.

The study’s specific inclusion criteria were as follows:

1. Confirmed GIS in thyroid ultrasonography and/or ^99^mTc scintigraphy during the neonatal period.
2. Thyroid autoantibodies were negative.
3. Patients who failed the trial-off of levothyroxine (LT_4_) at 3 years of age after CHT diagnosis, or even if successful, resumed and maintained LT_4_ several months or years later, owing to TSH abnormalities, or patients considering LT_4_ discontinuation.
4. The patients who underwent Next-Generation Sequencing (NGS) analysis who met the above criteria.

Patients with congenital abnormalities or syndromes identified at or before birth were excluded from the study. As a result, 33 patients in total were enrolled. The research involved comparing TSH and free thyroxine (fT_4_) levels at the time of diagnosis for all participants. Additionally, TSH and fT_4_ levels, along with LT_4_ dosages at 3 years of age, were reviewed and analyzed. However, data for some patients transferred to our center after turning 3 years old were incomplete. These missing records were managed systematically to ensure precise analysis. The study also included an examination of NGS analysis and a comparison of clinical manifestations across different genetic variants, as reported in existing literature. It important to note that not all patients underwent immediate genetic testing upon diagnosis. For those on prolonged LT_4_ treatment or maintaining TSH levels post-medication discontinuation, clinical guidance was provided for treatment cessation. Furthermore, in cases where a patient’s sibling with a confirmed genetic variant was diagnosed with CH, single-gene sequencing of the identified variant in the proband was conducted. This approach allowed for the exploration of varying clinical presentations among family members sharing the same genetic variants. This study received approval from the Institutional Review Board of Inha University Hospital (Approval No. 2023-03-026-000), and additional informed consent was obtained from the parents for segregation analysis when necessary.

### 2.2. NGS analysis and variant interpretation

Genomic DNA was isolated from the peripheral blood leukocytes. In all 33 patients, 23 genes associated with CHT were sequenced using NGS, and all coding exons and near-intron region-related genes were analyzed. The genetic panel test for hypothyroidism conducted at our institution included the genes *DUOX2*, *DUOXA2*, *FOXE1*, *GNAS*, *HESX1*, *IYD*, *LHX3*, *NKX2-1*, *NKX2-5*, *PAX8*, *POU1F1*, *PROP1*, *SCL16A2*, *SLC26A4*, *SLC5A5*, *TG*, *THRA*, *THRB*, *TPO*, *TRH*, *TRHR*, *TSHB*, and *TSHR.* Variants detected were interpreted and classified according to the American College of Medical Genetics and Genomics/Association for Molecular Pathology standards and guidelines.^[12]^ Variants with a minor allele frequency (MAF) >1% were removed based on various databases, such as GnomAD, ExAC, the 1000 Genomes Project, and KRGDB. Variants detected in the patients were confirmed using Sanger sequencing. Additionally, a genetic segregation study was performed using targeted variant sequencing of specific families.

### 2.3. Statistical analysis

Chi-squared tests of association were used to evaluate clinical data, and the Kruskal–Wallis test was used to evaluate differences in medians for each clinical parameter, hormone level, and dose of LT_4_. Statistical analyses were performed using SPSS version 25.0 (IBM, Armonk, NY), and results are presented as median (mean ± SD) or mean ± SD. Statistical significance was set at *P* < .05.

## 3. Results

In 33 patients enrolled in the study, the mean age of the patients was 10.15 years. The ratio of the number of boys to girls was 1.75:1 (21:12). All patients were born to non-consanguineous parents. The median TSH level at diagnosis was 50.34 (102.55 ± 136.77) mIU/L, and the median fT_4_ level was 0.88 (0.83 ± 0.44) ng/dL. The patients’ first serum blood test was performed at a median 11 (12.56 ± 6.30) days after birth. Six patients were born prematurely; their median age at birth was 30.5 (30.67 ± 2.42) weeks and their median TSH level was 36.94 (78.28 ± 225.84) mIU/L. The standard range of TSH around the 14th day of life in preterm babies is 4.9 ± 11.2 mIU/L for premature infants between 28 and 30 weeks of gestational age and 3.8 ± 9.3 mIU/L for premature infants between 31 and 34 weeks of gestational age.^[13]^ Additionally, their median fT_4_ level at diagnosis was 0.82 (0.75 ± 0.24) ng/dL.

In a study involving 33 patients, a total of 4 patients had siblings who were diagnosed with CHT. These siblings underwent single gene sequencing for the genetic variants identified in each respective proband.

### 3.1. NGS analysis and genetic variants

Among the 33 patients who underwent NGS analysis, genetic variants were identified in 26. (Table 1). The most prevalent variant detected in NGS analysis was in *DUOX2* (7/26, 26.92%) followed by *TSHR* (8/26, 30.77%). Variants in *TG* were found in 6 patients (6/26, 19.35%), whereas *DUOXA2* variants were observed in 5 (5/26, 19.23%). Additionally, variants in at least one *GLIS3* allele were confirmed in 3 patients (3/26, 11.54%), and an *NKX2-1* variant was identified in 2 patients (2/26, 7.69%). Each of *SLC26A4*, *SLC5A5*, *TRH*, and *THRB* had variants confirmed in 1 patient each (1/26, 3.85%). The numbers of patients represent those with at least 1 variant of the respective gene. No significant sequence variants were detected for *FOXE1*, *GNAS*, *HESX1*, *IYD*, *LHX3*, *NKX2-5*, *PAX8*, *POU1F1*, *PROP1*, *SCL16A2*, *THRA*, *TRHR*, or *TSHB*. Of the 26 patients with variants, 21 had a confirmed monogenic variant (4 in *DUOX2*, 3 in *DUOXA2*, 6 in *TSHR*, 4 in *TG*, 1 in *SCL5A5*, 1 in *GLIS3*, and 1 in *NKX2-1*). Additionally, 5 patients were diagnosed with a digenic variant (one each in *TSHR* and *DUOX2*, *TRH* and *DUOX2*, *GLIS3* and *DUOXA2*, *TG* and *GLIS3*, and *SLC26A4* and *NKX2-1*), and 1 patient had an oligogenic variant involving *DUOX2, TPO*, and *THRB*. Among them, patients #1, #5, #6, and #21 had siblings diagnosed CHT. Patient #1 had an affected identical twin brothers, and the other 3 patients had 1 affected sibling. Single gene analysis was done for each of those probands, and the variant was established on their sequence. The same variant had been detected on each of the siblings. Patients #1, #5, #6, and #21’s siblings are labeled in Table 3 as #1-1, #1-2, #5-1, #6-1, and #21-1, respectively, based on each proband’s number.

**Table 1:** Variants associated with congenital hypothyroidism and thyroid gland in situ.

Patient no.	Sex/age	Gene	DNA variant	Amino acid variation	Zygosity	Type of variant	Pathogenicity	Clinical type
1	F/9	*DUOX2* *DUOX2*	c.1883del c.3061C>T	p.(Lys628Argfs*11) p.(Arg1021*)	CompH	FS NS	PV LPV	PCH
2	F/16	*DUOXA2* *DUOXA2*	c.413dupA c.535T>C	p.(Tyr138Ter) p.(Tyr179His)	CompH	NS MS	PV LPV	PCH
3	F/6	*DUOX2*	c.1462G>A	p.Gly488Arg	Hom	MS	PV	PCH
4	F/18	*DUOX2* *TPO* *THRB*	c.1871del c.2787del c.122G>A	p.(Gly624Alafs*15) p.Met921fs -	Het Hom Het	FS FS MS	LPV VUS VUS	ACH
5	M/13	*TG* *TG*	c.2338C>T c.5548 + 1G>A	p.(Gln780Ter) -	CompH	NS SE	LPV LPV	PCH
6	F/12	*TSHR*	c.1349G>A	p.Arg450His	Hom	MS	PV	PCH
7	M/12	*TSHR*	c.1555C>T	p.Arg519Cys	Het	MS	PV	ACH
8	M/10	*TSHR*	c.1349G>A	p.Arg450His	Hom	MS	PV	ACH
9	M/5	*TSHR* *TSHR*	c.1349G>A c.242T>A	p.Arg450His p.Ile81Asn	CompH	MS MS	PV VUS	PCH
10	F/13	*TSHR* *DUOX2* *DUOX2*	c.734G>C c.4408C>T c.2560 + 4G>A	p.Gly245Ala p.Ala1470Trp -	Het CompH	MS MS SE	VUS VUS VUS	ACH
11	M/12	*TG* *TG*	c.2588T >C c.4746C>T	p.Leu863Pro p.Asp1582=	CompH	MS SE	VUS VUS	PCH
12	M/11	*NKX2-1*	c.1024G>A	p.Gly342Ser	Het	MS	VUS	TCH
13	F/5	*TG*	c.7783G>T	p.Aso2595Tyr	Het	MS	VUS	PCH
14	F/8	*DUOX2* *TRH*	c.3179C>T c.45C>A	p.Ala1060Val p.Asn15Lys	Hom Het	MS MS	VUS VUS	PCH
15	F/15	*SLC5A5*	c.1058 + 1G>A c.1060A>C	- p.Thr354Pro	CompH	SE MS	LP LP	PCH
16	M/11	*N/D*						ACH
17	M/10	*N/D*						ACH
18	M/12	*GLIS3* *DUOXA2*	c.554A>G c.535T>C	p.Asn185Ser p.Tyr179His	Het Het	MS MS	VUS VUS	ACH
19	M/14	*N/D*						TCH
20	M/14	*N/D*						PCH
21	M/9	*DUOXA2*	c.413dupA	pTyr138Ter	Hom	MS	PV	PCH
22	F/14	*TSHR*	c.1349G>A	p.Arg450His	Het	MS	PV	ACH
23	M/9	*N/D*						TCH
24	M/4	*TG* *GLIS3*	c.6559C>G c.1544C>G	p.Pro2187Ala p.Pro515Arg	Het Het	MS MS	VUS VUS	ACH
25	M/4	*TG*	c.2435C>T	p.Ala812Val	Het	MS	VUS	ACH
26	M/4	*DUOX2*	c.4375G>A	p.Asp1459Asn	Het	MS		TCH
27	F/15	*DUOXA2* *DUOXA2*	c.413dup c.738C>G	p.Tyr138* p.Tyr246*	CompH	NS NS	PV PV	TCH
28	M/4	*TSHR*	c.733G>A	p.Gly245Se	Hom	MS	VUS	PCH
29	M/13	*N/D*						ACH
30	M/9	*GLIS3*	c.951G>T	p.Glu317Asp	Hom	MS	VUS	ACH
31	F/7	*DUOX2* *DUOX2*	c.605_621del c.1462G>A	p.Gln202Argfs* p.Gly488Arg	CompH	FS MS	PV PV	PCH
32	M/3	*N/D*						TCH
33	M/14	*SLC26A4* *NKX2-1*	c.2168A>G c.953G>A	p.His723Arg p.Gly318Asp	Het	MS MS	PV VUS	TCH

ACH = ambiguous congenital hypothyroidism, CompH = compound heterozygous, FS = frameshift, Het = heterozygous, Hom = homozygous, LPV = likely pathogenic variant, MS = missense, NS = nonsense, PCH = permanent congenital hypothyroidism, PV = pathogenic variant, SE = splicing error, TCH = transient congenital hypothyroidism, VUS = variant of uncertain significance.

**Table 2:** Clinical and laboratory characteristics of the patients.

Parameter	PCH (n = 18)	ACH (n = 14)	TCH (n = 6)	*P* value
Sex: male/female	8/10	9/5	5/1	.99
Gestational age (weeks)	37.19 ± 34.10	36.85 ± 38.70	37.80 ± 1.92	.99
Birth weight (g)	2913.44 ± 694.74	2992.31 ± 845.19	2806 ± 548.80	.50
Prematurity	6	1	1	.99
Initial serum TSH	158.95 ± 190.62	60.66 ± 78.57	62.07 ± 26.15	.37
Initial fT_4_	0.76 ± 0.44	0.95 ± 0.46	0.75 ± 0.41	.48
LT_4_ dose at 3 years old	3.48 ± 0.72	2.71 ± 0.97	1.44 ± 0.36	.001
Current LT_4_ dose or at the time of withdrawal	1.76 ± 0.57	1.35 ± 0.58	1.44 ± 0.36	.31

ACH = ambiguous congenital hypothyroidism, fT_4_ = free thyroxine, LT_4_ = levothyroxine, PCH = persistent congenital hypothyroidism, RR = reference range, TCH = transient congenital hypothyroidism, TSH = thyroid-stimulating hormone.

**Table 3:** Variants and clinical characteristics of each group.

	TSH ≥ 50						TSH < 50					
fT_4_ < 0.89	Patient no.	TSH (mIU/L)	fT_4_ (ng/dL)	Variant	Type of variant	Clinical type	Patient No.	TSH (mIU/L)	fT_4_ (ng/dL)	Variant	Type of Variant	Clinical type
	1	483	0.4	DUOX2	CompH	PCH	20	25.83	0.76	N/D		PCH
	2	57	0.87	DUOXA2	CompH	PCH	23	46.67	0.88	N/D		TCH
	3	150	0.17	DUOX2	Hom	PCH						
	5	>500	0.06	TG	CompH	PCH						
	5-1	>500	<0.04	TG	CompH	PCH						
	13	150	0.80	TG	Het	PCH						
	14	50.34	0.65	DUOX2 TRH	Hom Het	PCH						
	15	393	0.26	SLC5A5	CompH	PCH						
	31	305	0.31	DUOX2	CompH	PCH						
	4	50	0.04	DUOX2 TPO THRB	Het Hom Het	ACH						
	17	63.7	0.75	N/D		ACH						
	30	58.5	0.76	GLIS3	Hom	ACH						
	19	100	0.18	N/D		TCH						
	32	71.1	0.52	N/D		TCH						
fT_4_ 0.89–2.2	1–2	57.9	0.92	DUOX2	CompH	PCH	1-1	26.3	1.37	DUOX2	CompH	PCH
	9	60.77	1.2	TSHR	CompH	PCH	6	15.65	1.06	TSHR	Hom	PCH
	25	50.5	1.34	TG	Het	ACH	6-1	23.23	0.92	TSHR	Hom	PCH
	26	61.8	0.93	DUOX2	Het	TCH	11	37.10	1.17	TG	CompH	PCH
							28	13.06	1.39	TSHR	Hom	PCH
							7	14.66	0.89	TSHR	Het	ACH
							8	23.64	1.26	TSHR	Hom	ACH
							16	20.36	1.46	N/D		ACH
							18	40.80	1.09	DUOXA2 GLIS-3	Het Het	ACH
							22	27.20	0.99	TSHR	Het	ACH
							24	47	0.92	TG GLIS-3	Het Het	ACH
							29	26.50	1.62	N/D		ACH
							12	30.8	1.26	NKX2-1	Het	TCH

ACH = ambiguous congenital hypothyroidism, CompH = compound heterozygote, fT4 = free thyroxine, Het = heterozygote, Hom = homozygote, PCH = permanent congenital hypothyroidism, TCH = transient congenital hypothyroidism, TSH = thyroid stimulating hormone.

### 3.2. Clinical classification: permanent, transient, and ambiguous hypothyroidism

We divided the 38 patients including 5 siblings into 3 groups—transient congenital hypothyroidism (TCH), permanent congenital hypothyroidism (PCH), and ambiguous congenital hypothyroidism (ACH)—according to the results of the LT_4_ discontinuation attempt at 3 years of age. After the first LT_4_ trial-off around the age of 3 years, the TSH value remained between 5 and 10 mIU/L for several months but normalized over time while being monitored without medication. Patients who initially experienced a failed trial-off but eventually had a successful trial-off before the age of 5 years were also classified into the TCH group. Patients who, within 3 months after attempting medication, experienced a re-elevation of their TSH levels to >10 mIU/L and had to resume LT_4_ treatment were classified as having PCH. In cases where genetic testing was conducted before the age of 3 years and a variant was identified, a trial-off was not performed. Additionally, if the proband’s sibling, who had the same variants, was continuously receiving LT_4_ for PCH, a trial-off was not performed. All 10 patients were grouped and classified as having PCH. We categorized patients with ACH as those who, after the medication discontinuation attempt, had TSH levels between 5 and 10 mIU/L while maintaining a normal fT_4_ level. In addition, some patients sustained normal thyroid function test (TFT) results for a certain period after the discontinuation of LT4. However, a blood test conducted several months or years later revealed a TSH level exceeding 10 mIU/L, whereas fT_4_ levels remained within the normal range. In these cases, LT_4_ treatment was temporarily resumed and discontinued. These patients were categorized as having ACH. The results indicated that 6, 18, and 14 patients were classified into the TCH, PCH, and ACH groups, respectively (Table 2). In 3 groups, the sex distribution characterized a considerable but not statistically significant predominance of males in the considered groups compared to the TCH group, where the distribution of sex is almost uniform. It characterized the high prevalence of prematurity in PCH group, in comparison with ACH and TCH. However, it showed that the gestational age and birth weight were almost similar among the groups; hence, as born, the parameters considered showed no difference among the 3 groups. The mean initial serum TSH levels showed significantly high in PCH group, indicating birth with more serious dysfunction of thyroid. However, there was no statistical difference in fT_4_ level among the groups at the initial level. The LT_4_ required dosage at 3 years was significantly high in the PCH group. Additionally, our analysis identified several patients with oligogenic variants. However, the clinical outcomes for these patients varied widely. For instance, patient #33, with heterozygous variants in *SCL26A4* and *NKX2-1*, categorized as TCH, suggesting that the presence of multiple variants did not necessarily correlate with a more severe phenotype. Conversely, patient #5 with compound heterozygous TG variants exhibited PCH. These findings indicate that while oligogenic variants can theoretically lead to a compounded loss of enzyme activity, the clinical impact may be moderated by other biological factors, such as residual enzyme activity or compensatory pathways.

### 3.3. Differences in clinical characteristics depending on initial TSH levels

**Figure 1.**
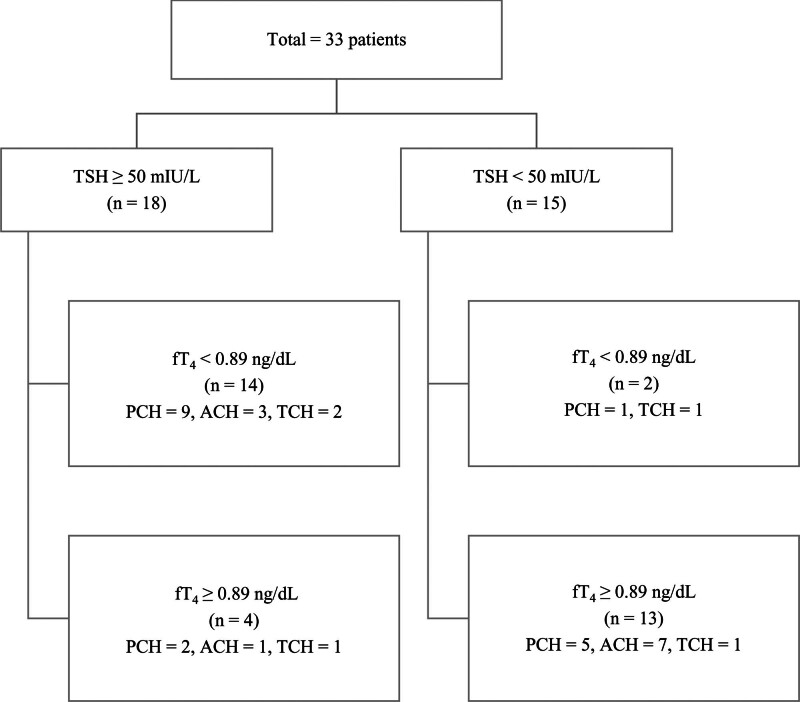
Subgrouping based on initial thyroid stimulating hormone and free thyroxine levels. This displays a scatter plot categorizing 33 patients and 5 siblings based on their initial thyroid-stimulating hormone (TSH) and free thyroxine (fT_4_) levels at diagnosis of congenital hypothyroidism. Each point represents an individual patient, grouped into quadrants to illustrate distinct initial diagnostic categories. Additionally, the plot schematically shows the distribution of patients classified as having persistent, ambiguous, or transient hypothyroidism. The number of patients in each category is indicated, providing a visual overview of the initial thyroid function status across the cohort. ACH = ambiguous congenital hypothyroidism; fT_4_ = free thyroxine; PCH = persistent congenital hypothyroidism; TCH = transient congenital hypothyroidism; TSH = thyroid-stimulating hormone.

We divided the patient groups according to the TSH value at the time of diagnosis, and the group with TSH > 50 mIU/L was subdivided into those with fT_4_ < 0.89 ng/dL at diagnosis and those with normal fT_4_ levels (reference: 0.89–2.2 ng/mL) (Fig. 1). We collectively examined and analyzed the clinical characteristics and genetic variants (Table 3). Among the 38 patients, 18 had serum TSH > 50 mIU/L at the time of diagnosis and 15 had serum TSH < 50 mIU/L. The TSH levels in the 2 groups were significantly different (*P* < .001). Since 5 patients were transferred to our hospital from another medical institution and some succeeded in discontinuing LT_4_ in the past but unintentionally visited our hospital, an incidental elevated TSH level was confirmed through a blood test. Thus, the TSH levels at the time of initial diagnosis were not available.

### 3.4. TSH levels > 50 mIU/L and low fT4 levels

Among the 18 patients with TSH > 50 mIU/L, 14 had low fT_4_ levels (<0.89 ng/dL). Within this group, 9 patients were categorized as having PCH, 3 as having ACH, and 2 as having TCH. Of the 9 patients classified as having PCH, 8 had homozygous or compound heterozygous variants (4 for *DUOX2*, 1 for *DUOXA2*, 2 for *TG*, and 1 for *SLC5A5*). One patient harbored a heterozygous *TG* variant. Genetic variants were identified in 2 of the 3 patients classified as having ACH: one had an oligogenic variant and the other had a homozygous *GLIS3* variant. Notably, within this group, neither of the 2 patients with a clinical course of TCH exhibited any genetic variants.

### 3.5. TSH levels > 50 mIU/L and normal fT4 levels

Among the 4 patients with TSH > 50 mIU/L and fT_4_ levels within the normal range, 2 exhibited PCH. They showed compound heterozygous variants in both *DUOX2* and *TSHR*. One patient classified as having ACH had a heterozygous *TG* variant, and 1 patient categorized as having TCH had a heterozygous *DUOX2* variant.

### 3.6. TSH levels < 50 mIU/L

Of the 15 patients in the group with TSH < 50 mIU/L, 13 exhibited normal fT_4_ levels. In this group, there were 6 patients with PCH, 7 with ACH, and 2 with TCH. Among these, 11 patients had genetic variants. In this subgroup, similar to the previous group, compound heterozygous or homozygous *DUOX2, TSHR,* and *TG* variants were confirmed in 5 of the 6 patients classified as having PCH. Five patients with ACH harbored genetic variants. Among them, one had a homozygous *TSHR* variant and 4 had heterozygous *TSHR*, *GLIS-3*, *DUOXA2*, and *TG* variants. In 1 of the 2 patients exhibiting TCH, a heterozygous *NKX2-1* variant was identified.

### 3.7. Long-term follow-up monitoring of goiter

During follow-up, 2 patients were found to have goiters. Patient #4 exhibited variants in *DUOX2* and *THRB*, with a homozygous *TPO* variant. The patient was diagnosed with a nodular goiter and underwent regular monitoring using periodic ultrasonography (US) and fine-needle biopsy (FNA). Patient #15 had a compound heterozygous *SLC5A5* variant. While monitoring the patient’s medication progress, a visibly enlarged nodular goiter was observed at 14 years of age. On the basis of the US and FNA results, malignancy could not be ruled out, and the patient was transferred to the Department of General Surgery. She underwent surgical thyroidectomy, and the final biopsy confirmed a thyroid follicular adenoma.

## 4. Discussion

Among the 31 patients with confirmed genetic variants in this study, the most frequently identified variants were in *DUOX2*, *TSHR*, *TG*, and *DUOXA2*, in the given order. Similarly, in previous studies conducted in South Korea, Japan, China, and the United Kingdom, *DUOX2* variants were predominant in these populations.^[14–18]^ *DUOX2* and *TSHR* variants are known to present various phenotypes ranging from euthyroid to hyperthyrotropinemia in children, depending on the genotype, race, and region.^[18–22]^

Many countries conduct screening tests for CHT, resulting in a rapid diagnosis and appropriate treatment for numerous patients, thus preventing neurodevelopmental, growth, and behavioral problems associated with untreated CHT. In general, CHT with GIS patients are at risk of developing PCH if their TSH level remains above 10 mIU/L despite LT_4_ treatment at approximately 1 year of age, or if the LT_4_ dosage continues to increase during the course of treatment until 3 years of age.^[23]^ Nonetheless, the TSH level at the time of diagnosis alone cannot predict the prognosis of patients with CHT, including whether they will transition to permanent or transient CHT.^[23,24]^ In real clinical scenarios, clinicians frequently encounter the dilemma of determining the duration for which they should continue prescribing LT_4_ to patients with CHT. Specifically, when TSH and fT_4_ levels are well maintained on LT_4_ and the dosage remains consistent or is reduced, additional medical tools, including the results of a trial-off conducted at 3 years of age, may be necessary to make a decision regarding the discontinuation of LT_4_. Therefore, considering the results of this study, genetic analysis can be used not only to reveal the etiology of CHT but also to determine the subsequent treatment course and predict prognosis. This holds true even in cases where the typical 3-year-old trial-off, along with TSH, fT_4_, and LT_4_ dosages, proves ineffective as a reliable clinical indicator.

According to previous findings, 40% of infants diagnosed with CHT at birth are subsequently diagnosed with PCH.^[23,25]^ One study reported that 54% of patients initially treated for CHT with GIS during the neonatal period had a transient form of the condition.^[26]^ In our study, when considering the overall results, 18 patients (18/38, 47.37%) experienced failure or encountered challenges during trial-off, and this outcome did not differ significantly from previous research findings. However, among the studies conducted so far, no clear cohort study or large-scale study has been conducted on the diagnosis rate of PCH or TCH in patients with CHT accompanied by a genetic variant. Therefore, in this study, patients were subdivided according to TSH levels, which are commonly used for the initial diagnosis of CHT, and further divided into subgroups based on fT_4_ levels. Their genetic variant reports were analyzed along with the clinical course observed after the age of 3 years, employing a method not previously attempted in similar studies. Interestingly, as shown in Table 3, among patients with high initial TSH and low fT_4_ levels, those treated with compound heterozygous or homozygous variants had a high tendency to fail the trial-off and transition to PCH. Within this group, patients with compound heterozygous or homozygous *DUOX2/DUOXA2* and *TG* variants predominantly displayed clinical signs of PCH. In contrast, individuals with heterozygous variants in *DUOX2* and *TG* tended to exhibit ACH features. Interestingly, although Patient #1 and her siblings had the same gene variants, their TSH and fT_4_ levels at the time of initial diagnosis were significantly different. This may be attributed to the fact that, unlike Patient #1, who was born full-term and underwent a serum TSH test at approximately 2 weeks of age, his younger siblings were premature infants born at 36 weeks of age. Furthermore, the relatively rapid performance of the serum TSH and fT_4_ tests was facilitated by the known medical history of Patient #1. Studies on the prognosis of *DUOX2* variants vary by race and country; however, CHT caused by *DUOX2* variants manifests as mild CH and tends to be transient.^[14,16,27]^ Compared to *DUOX2*, *TG* variants cause consistent PCH with a spectrum of various clinical presentations.^[28]^ Meanwhile, in the patient group with initial TSH < 50 mIU/L at the time of diagnosis, there were relatively many patients with *TSHR* variants; their clinical manifestations were diverse and they were classified as either PCH or ACH. Among the 7 patients with *TSHR* variants, all 6, except Patient #9, had TSH levels < 50 mIU/L at diagnosis and all fT_4_ levels were within the normal range. Patient #9 had normal fT_4_ levels, although with TSH > 50 mIU/L at diagnosis. Additionally, among patients with *TSHR* variants, the average dose of LT_4_ in the 6 patients currently under treatment was 1.76 ± 0.84 μg/kg, which is lower than the LT_4_ dose of 2.22 ± 0.52 μg/kg reported in a study conducted in Japan on patients with *TSHR* variants.^[29]^

In contrast to Patient #4, who presented with oligogenic variants of *DUOX2, THRB*, and *TPO* and underwent continuous follow-up owing to a simple goiter, Patient #15 had an enlarged nodular goiter and was finally diagnosed with thyroid follicular adenoma. *SLC5A5* is not only well known for its association with CHT but is also well known as a gene that can predict the possibility of cancer based on a blood test before thyroid cancer surgery.^[30,31]^ In particular, *SLC5A5* has a high correlation with thyroid follicular adenoma but not with carcinoma.^[31]^ Early diagnosis is crucial because endocrine tumors are often diagnosed as carcinomas based on the extent of invasion into the surrounding tissues rather than the direct presence of malignant cells. Goiters tend to occur in patients with *TG*, *TPO*, and *DUOX2* variants, whereas nodules are particularly common in patients with *TG* and *TPO* variants.^[9,28,32]^ Periodic US is necessary for CHT with GIS patient who present with goiters. Moreover, our analysis of these 2 patients with goiter confirmed that even when similar CHT with GIS patients exhibit goiter progression, disease advancement may differ depending on the causal genetic variant. These findings imply that the use of genetic analysis as an additional diagnostic tool is highly significant.

Our findings suggest that while oligogenic variants in CHT can potentially lead to a “cross-loss of enzyme activity,”^[7]^ this does not uniformly predict more severe clinical outcomes. The clinical variability observed in patients with multiple gene variants could have the need for a nuanced approach to genetic testing and patient management. Personalized treatment strategies should consider the individual genetic makeup, potential compensatory mechanisms, and overall clinical context to optimize care for CHT patients.

Although previous research on genetic variants linked to CHT has explored aspects such as variant prevalence in cohorts and the clinical presentations associated with each variant, there remains a lack of studies on how genetic analysis results can inform the discontinuation of LT_4_ in patients with confirmed genetic variants in CHT. Drawing the conclusion from our study, it was evidenced that genetic analysis would significantly define strategies concerning monitoring and management in CHT with GIS patients. This is especially likely in a patient genetically confirmed to have heterozygous variants in *DUOX2*, *DUOXA2*, and heterozygous or homozygous *TSHR*, where a mild increase in TSH was borderline at birth. In such a scenario, the approach to LT_4_ therapy may be individualized based on the genetic test with conventional blood tests. Genetic information would allow for a more exact decision on the duration of LT_4_ therapy. This is because it allows an individualized regime of treatment based on the idiosyncrasies in the genetic makeup of each patient and keeps in mind the time-bound thyroid function. In fact, it will allow overtreatment to be escaped from the patients in whom once investigated, the genetic profile could report likely transient character of CHT if prolonged hormone therapy, focusing on the balancing and the concern of the possible collateral effects. This approach in practice, therefore, could allow for improved care in that the treatment and monitoring are not, acutely, clinically guided based on symptoms or biochemical test results but rather genetically informed. Targeted strategy monitoring ensures that timely and proper intervention is done: optimized outcome of patients with CHT, efficiency in resource use in healthcare. Principles to make the study’s findings more tailored, effective, and nuanced in the management of this disorder. In this regard, we anticipate that this study, in conjunction with the blood and imaging test results outlined in previous studies, will prove beneficial for patients with CHT in making treatment plans and strategies for the discontinuation of LT_4_ in real-world clinical settings. We anticipate that future larger cohort studies and more refined research will facilitate in-depth discussions on the appropriate use of genetic analyses to manage CHT.

### 4.1. Limitations

This was a limited retrospective study with a small cohort of registered patients, which was insufficient to ascertain statistical differences in clinical parameters. Moreover, it was difficult to retrospectively examine the medical records of patients who were not born at our hospital. Primarily, owing to the high incidence of trial-off failures around the age of 3 years, persistent subclinical hypothyroidism among registered patients, and the inclusion of numerous siblings, there is a possibility of a selection bias in the variant detection rate.

## 5. Conclusions

NGS analysis of CHT is useful for identifying the genetic causes of the disease. In this study, we found that *DUOX2*, *TSHR*, *TG*, and *DUOXA2* variants were dominant in CHT with GIS patients at a single center. Previous studies have primarily focused on the types and prevalence of genetic variants associated with DH and their clinical characteristics. However, in this study, we aimed to conduct a comprehensive analysis of the clinical patterns associated with each genetic variant of TFT and to contemplate the clinical utilization of genetic analysis results for CHT treatment. According to the current treatment guidelines for CHT, decisions regarding the discontinuation and resumption of LT_4_ are based on TFT or LT_4_ doses at the age of 3 years.^[4]^ Our results confirmed that initial biochemical findings generally demonstrate mild values in CHT with GIS patients with confirmed *TSHR* variants. In addition, variable clinical characteristics have been observed in patients with *DUOX2* variants. These findings suggest that genetic testing can predict patient prognosis and treatment progress. It is necessary to conduct long-term follow-up studies on the clinical progression of CHT in patients with *DUOX2* and *TSHR* variants. Further studies are required to assess the effectiveness of LT_4_ therapy in patients with persistent hyperthyrotropinemia. Patients with goiter variants should undergo regular radiological monitoring. Finally, large-scale studies are warranted to investigate the effective utilization of genetic analysis in treatment of patients with CHT.

## Acknowledgments

We express our deepest gratitude to the children diagnosed with congenital hypothyroidism at a very early age and to their parents who care for them with love and dedication.

## Author contributions

**Conceptualization:** Jisun Park, Su-Jin Kim, Ji-Eun Lee.

**Data curation:** Eun Young Joo, Myung Ji Yoo, Woori Jang.

**Formal analysis:** Jisun Park.

**Investigation:** Eun Young Joo.

**Validation:** Woori Jang.

**Writing – original draft:** Jisun Park.

**Writing – review & editing:** Jisun Park, Su-Jin Kim, Ji-Eun Lee.
